# Editorial: The Anatomical Basis of the Cross Talk Between Immune System and Brain

**DOI:** 10.3389/fnana.2020.00024

**Published:** 2020-05-08

**Authors:** Francesco Fornai, Francesco Orzi

**Affiliations:** ^1^Department of Translational Research and New Technologies in Medicine and Surgery, University of Pisa, Pisa, Italy; ^2^IRCCS Neuromed, Pozzilli, Italy; ^3^Department of Neuroscience, Mental Health and Sense Organs NESMOS, Sapienza University of Rome, Rome, Italy

**Keywords:** neuro-immunity, neuro-inflammation, neuro-immune system, neuro-immunological organelles, immunoproteasome

Innate and adaptive immunity are intensely connected with the central nervous system (CNS) including both spinal cord and the brain. Functional studies shed the basis to define a cross talk between the immune- system and the brain in previous decades. Nowadays a number of research manuscripts indicate a strong interaction between the immune system and neural cells involving both glia and neurons, which is evident also by studying the brain structure. Since most adaptive and innate immune cells reach out the brain through blood and lymphatic vessels, the interconnection recruits along with neurons and glia also the whole brain vessels and their innervation. Thus, the anatomical basis of the cross-talk between the immune system and the brain can be identified in each compartment hosted in the gray and white matter of the CNS. These anatomical connections are analyzed at multiple levels ranging from electron and light microscopy *ex vivo* in humans and experimental models, and through *in vivo* imaging in human beings as well as experimental settings, allowing MRI-based neuro-anatomical studies (Iadecola, 2017). Each anatomical compartment (namely: neurons, glia, blood, and lymphatic brain vessels), as well as their innervation from peripheral and central neuriter are taken into account in the present Research Topic. Apart from the analysis of each anatomical structure contributing to understand the interactions between the immune system and the brain, a supra-modal entity is defined by functional anatomy, which is known as neurovascular unit (Iadecola, 2017). In fact, evidence accumulates showing that focal areas of the brain work in synergy upon specific physiological and pathological conditions to orchestrate neurons glia blood supply and lymphatic draining aimed at providing the best micro-environment to sustain baseline brain activities, critical neurological challenges, as well as compensating from neurological insults. The integration within the neurovascular unit is triggered and kept finely tuned upon demand by multiple and synchronous innervation of each anatomical constituent of the unit itself. This is generated by peripheral nerves arising from the sphenopalatine ganglion (for the parasympathetic component) as well as vegetative nerves running in the external layer of brain arteries, which arise from the cervical ganglia of the orthosympathetic nervous system. This double innervation from the peripheral sympathetic nervous system grants the coordination of the blood flow with functional needs from rather large brain areas and it is substituted by a central innervation to tune focal brain sites which are nurtured by the smallest brain arteries. Such a central innervation within focal district is provided by norepinephrine-containing axons arising from a few nuclei of the brainstem, such as the locus coeruleus or the serotonin-containing axons arising from the dorsal raphe nucleus (Bucci et al., 2017). In this way, the paracrine effects induced by the release of these monoamines (Agnati et al., 1995; Agnati and Fuxe, 2000) may extend to the focal sites, which is responsible for the smallest neurovascular unit. This is addressed clearly in this Research Topic (Troili et al.), which identifies the perivascular unit as the space where the cross-talk between immune and nervous system, joined by brain vessels, takes place. Such a space, is believed to represent what was once defined as Wirchov-Robin space which is thought to be in anatomical connection with the meningeal lymphatics that ultimately drains the lymph flow outside the brain. This is why the imaging of the glymphatic spaces at the level of the dura mater allows to study the anatomical connection between nervous and immune system. The maturation of this space is described in this Research Topic (Bálint et al.), and its altered function may play a role in specific developmental disorders such as the posthemorrhagic hydrocephalus of prematurity, where the role of neuroinflammation was emphasized (Robinson et al.). The relevance of the glymphatic space is demonstrated in multiple sclerosis (Cavallari et al., 2018) and it is altered in specific neuroimmunological disorders reported in the present Research Topic, such as neuromyelitis optica (Nicolas et al.). Moreover, such an altered cross-talk is thought to play a role also in degenerative disorders, such as Alzheimer's disease (AD), cognitive impairment (Giorgi et al., 2017, 2019; Faraco et al., 2019) and stroke (Garcia-Bonilla et al., 2018). In fact, the anatomical intermingling between these structures may underlie the neurological intersection between inflammation, autoimmunity, infection, stroke, and degenerative dementia (Giorgi et al., 2019; Parikh et al., 2020). In line with this, the noradrenergic component of the innervation of the neurovascular unit is impaired when neuroinflammation occurs in the course of dementia (Giorgi et al., 2019). The molecular mechanisms bridging altered immunity and cognitive impairment were analyzed by specific contribution of the Research Topic (Cipollini et al.), showing that T helper 17 (Th17) cells, immune cells which are known to be important in the defense of the organism against extracellular infectious agents, are involved in cognitive impairment, multiple sclerosis, ischemic brain injury, and AD. In keeping with these molecular mechanisms, an article of the Research Topic discusses the mechanisms by which Th17 cells enter the brain and induce brain damage. This evidence emphasizes both direct and indirect mechanisms, including direct effects of IL-17 on brain cells and indirect effects due to disruption of the blood-brain barrier, neurovascular dysfunction and gut-brain axis. In keeping with molecular mechanisms, circulating nucleic acids play a fundamental role as shown here by an article (Gambardella et al.) disclosing how fragments of mitochondrial DNA (mtDNA) are released outside the cell and persist in extracellular fluids as circulating, cell-free, mtDNA (ccf-mtDNA). When compared to nuclear DNA, such a double stranded mtDNA is more resistant to nuclease degradation. The bridging between immunity to neuronal activity was further analyzed at intracellular level (Limanaqi et al.) showing how the ubiquitin proteasome may sustain communication between neurons, glia, and brain circulating T-lymphocytes both in baseline and pathological conditions. Upregulation of the immunoproteasome to the detriment of the standard proteasome, is implicated in the inflammation. Dysregulations of the immune-proteasome translate into altered communication between neurons, glia, and immune cells.

In summary, the Research Topic provided multi-level anatomical evidence concerning a bi-directional communication between nerve- and immune- cells showing that altered communication between the immune and nervous system is a common hallmark in neuro-developmental, neurodegenerative, and neuro-immunological diseases.

## Author Contributions

FF and FO equally contributed to writing and editing the editorial.

## Conflict of Interest

The authors declare that the research was conducted in the absence of any commercial or financial relationships that could be construed as a potential conflict of interest.
